# The role of GOT1 in cancer metabolism

**DOI:** 10.3389/fonc.2024.1519046

**Published:** 2024-12-24

**Authors:** Huan Peng, Huihong Dou, Sheng He, Yu-an Xie, Qinle Zhang, Jianqiu Zheng

**Affiliations:** ^1^ Birth Defects Prevention and Control Institute, Maternal and Child Health Hospital of Guangxi Zhuang Autonomous Region, Nanning, China; ^2^ Guangxi Key Laboratory of Reproductive Health and Birth Defect Prevention, Maternal and Child Health Hospital of Guangxi Zhuang Autonomous Region, Nanning, China; ^3^ Guangxi Clinical Research Center for Pediatric Diseases, Maternal and Child Health Hospital of Guangxi Zhuang Autonomous Region, Nanning, China; ^4^ Guangxi Key Laboratory of Birth Defects and Stem Cell Biobank, Maternal and Child Health Hospital of Guangxi Zhuang Autonomous Region, Nanning, China; ^5^ Guangxi Key Laboratory of Birth Defects Research and Prevention, Maternal and Child Health Hospital of Guangxi Zhuang Autonomous Region, Nanning, China

**Keywords:** cancer, cell metabolism, GOT1, therapeutic target, metabolic reprogramming

## Abstract

GOT1, a cytoplasmic glutamic oxaloacetic transaminase, plays a critical role in various metabolic pathways essential for cellular homeostasis and dysregulated metabolism. Recent studies have highlighted the significant plasticity and roles of GOT1 in metabolic reprogramming through participating in both classical and non-classical glutamine metabolism, glycolytic metabolism, and other metabolic pathways. This review summarizes emerging insights on the metabolic roles of GOT1 in cancer cells and emphasizes the response of cancer cells to altered metabolism when the expression of GOT1 is altered. We review how cancer cells repurpose cell intrinsic metabolism and their flexibility when GOT1 is inhibited and delineate the molecular mechanisms of GOT1’s interaction with specific oncogenes and regulators at multiple levels, including transcriptional and epigenetic regulation, which govern cellular growth and metabolism. These insights may provide new directions for cancer metabolism research and novel targets for cancer treatment.

## 1 Introduction

Cancers reprogram cellular metabolism to meet the bioenergetic, biosynthetic, and redox demands of malignant cells, supporting their aberrant proliferation and survival (1, 2). These reprogrammed activities are recognized as hallmarks of cancers, as many cancer types exhibit general metabolic alterations (2, 3). Normal cell metabolism generates energy for maintaining physiological activities and cellular functions through complex reactions (4). However, cancer cells reprogram metabolism to acquire necessary nutrients from a frequently nutrient-poor environment and repurpose these nutrients to sustain viability and build new biomass (1, 2, 5, 6). Understanding how metabolism is rewired in cancer cells, and utilizing these metabolic changes for therapeutic benefits are key research areas. Accruing evidence suggests that metabolic regulation plays a predominant role in determining cell states, including proliferation and senescence. Therefore, targeting cell metabolism or the key enzymes involved in these processes may be a new strategy for treating refractory cancers.

GOT1 is a key enzyme in cell metabolism, distributed in the liver, muscle, heart, kidney, brain, and other tissues (7). The *GOT1* gene, located on human chromosome 10q24.1–25, consists of 9 exons, and encodes a 46.2 kDa protein composed of 413 amino acids (8). GOT1 exhibits abnormal expression in numerous cancers. The expression of GOT1 was up-regulated in pancreatic ductal adenocarcinoma (PDAC) (9–11), colorectal cancer (12, 13), breast cancer (14–16), lung adenocarcinoma (17), glioblastoma (17), prostate cancer (17–19), acute myeloid leukemia (20), and multiple myeloma (21), while down-regulated in poorly-differentiated hepatocellular carcinoma cells (22). Aberrant expression of GOT1 serves as a candidate biomarker. In most cases, elevated expression of GOT1 in cancer is associated with poor prognosis (23, 24). For example, GOT1 expression could be served as an independent prognostic biomarker in PDAC (9). Additionally, serum GOT1 levels are indicators of liver dysfunction in both liver and non-liver tumors, cardiovascular diseases, type 2 diabetes, and all-cause mortality (25–31).

**Figure 1:**
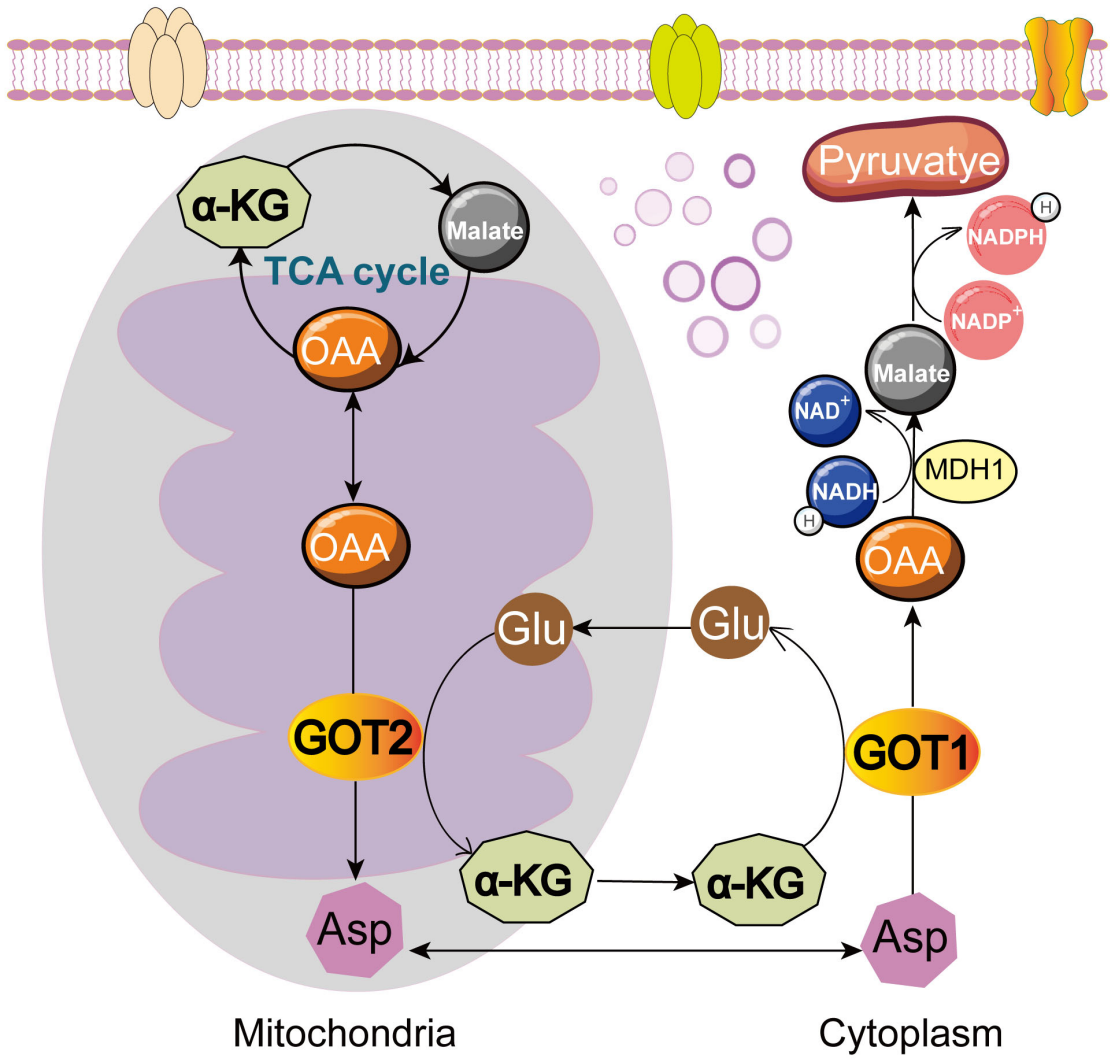
Roles of GOT1 and GOT2 in cell metabolism. GOT1 catalyzes Asp and α-KG into OAA and Glu in cytoplasm. OAA is further converted to malate by MDH1, and then to pyruvate, producing NADPH. GOT2 reversibly catalyzes OAA and Glu into Asp and α-KG, fueling TCA cycle. Glu, glutamate; Asp, aspartate; OAA, oxaloacetate; α-KG, α-ketoglutarate; MDH1, malate dehydrogenase 1.

Cytoplasmic GOT1 and its mitochondrial isozyme GOT2 usually occur together and interact with each other in metabolic processes (32–34). GOT1 is mainly present in the cytoplasm and GOT2 exists in the mitochondrial matrix. GOT1 catalyzes aspartate (Asp) and α-ketoglutarate (α-KG) into oxaloacetate (OAA) and glutamate (Glu) in cytoplasm. Then, Glu can generate Asp or be metabolized into other products through tricarboxylic acid (TCA) cycle. Glu can be converted into α-KG via the deamination of glutamate dehydrogenase 1 (GLUD1) and catalyzed to generate Asp by GOT2 in mitochondrial matrix. And then Asp from mitochondrial matrix is converted to OAA via GOT1 in cytoplasm (35). OAA is catalyzed by malate dehydrogenase 1 (MDH1) to generate malate, which is catalyzed to pyruvate through malic enzyme (ME). In this process, NADPH is produced and NADPH/NADP^+^ ratio is increased to maintain reactive oxygen species (ROS) balance (36). GOT2 reversibly catalyzes OAA and Glu into Asp and α-KG, contributing to the TCA cycle and energy production (37–43) (**Figure 1**). Both GOT1 and GOT2 are crucial components of the malate-aspartate shuttle (MAS), transferring reducing equivalents from NADH into the mitochondria for oxidative phosphorylation (44). Cancer cells utilize the amino acid Glu to support the anabolic processes and promote cell growth. The expression of GOT1 could be regulated by oncogene KRAS (10, 45) or other regulators (43, 45–47) like non-coding RNAs (24, 48–52), affecting Asp synthesis, NADPH production, glucose metabolism, as well as other metabolic pathways, and then tumor cells rewired metabolism to support their proliferation. This process links the expression of GOT1 closely to cancer progression. This review will dissect GOT1-related metabolic mechanisms and the molecular pathways about how GOT1 influences cancer progression. We also discuss potential limitations, challenges, and practical implications in targeting GOT1 therapeutically.

## 2 Main functions of GOT1 in cancer

**Figure 2:**
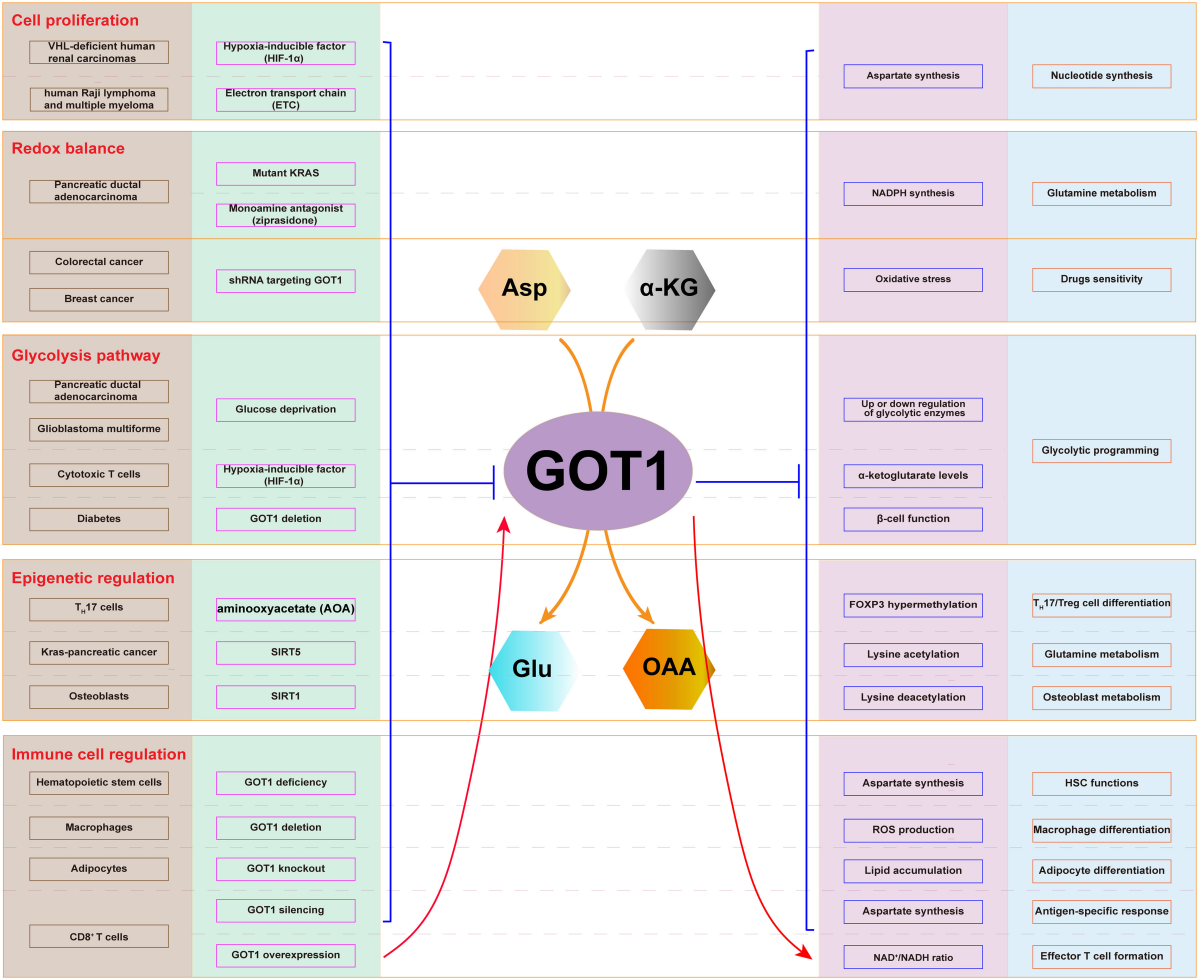
Cellular metabolic pathway involving GOT1. The expression of GOT1 is regulated by HIF-1α and ETC, which affects Asp synthesis and nucleotide production in cancer cells. Mutant KRAS and ziprasidone regulate the expression of GOT1, which influence NADPH production, oxidative stress, and Gln metabolism. GOT1 participates in glycolysis upon glucose deprivation. GOT1 participates in epigenetic regulation through lysine acetylation and deacetylation. GOT1 is involved in hematopoietic stem cell and T cell function, as well as T cell, macrophage, and adipocyte differentiation.

Cancer cells utilize Asp and glutamine (Gln) to support their metabolic process, producing energy, and promote cell proliferation. GOT1, involved in this process, participates in several critical metabolic functions: Asp synthesis, Gln metabolism, glycolysis pathway regulation, immune cell function regulation, and epigenetic regulation (**Figure 2**). Therefore, GOT1 has become a key consideration when interrogating cancer metabolism.

### 2.1 Supply of Asp to support cell proliferation

The deregulated cell growth and division in cancers imposed more requirements for DNA. Asp, a vital rate-limiting product of GOT1-catalyzed reversible reaction, is crucial for cancer cells due to its roles in nucleotide synthesis. Cellular Asp drives cancer cell proliferation. Disruption of GOT1 significantly impacts Asp synthesis and consequently affects cancer cell growth. For example, the expression of GOT1 was suppressed by hypoxia-inducible factor-1α (HIF-1α) in VHL-deficient human renal carcinoma (43), and by electron transport chain (ETC) in human Raji lymphoma and KMS-26 multiple myeloma (38, 53), leading to impaired proliferation of cancer cells. Mechanistically, the abnormal proliferation of these tumor cells was due to the reduction of Asp synthesis through inhibiting Gln oxidation and attenuating reductive carboxylation following GOT1 deletion, which disrupted nucleotide generation within the cells. Additionally, GOT1 treatment reduced serum Glu concentration, and GOT1-induced protective effects in cerebral ischemia were mediated by Asp metabolism (54).

### 2.2 Regulation of Gln metabolism to maintain redox homeostasis

GOT1 is crucial for producing NADPH and maintaining redox homeostasis through Gln metabolism. Emerging studies showed that in PDAC, KRAS knockdown (10, 13, 55) or the use of selective monoamine antagonist ziprasidone (56, 57) directly targeted and inhibited the expression of GOT1, subsequently disrupting Gln metabolism, affecting NADPH production, elevating ROS levels, and disturbing the redox balance. Mechanistically, there are some differences between the two approaches. PDAC relied on a distinct KRAS-regulated metabolic pathway to fuel the TCA cycle. KRAS activated the expression of GOT1. At this moment, Gln-derived Glu was converted to Asp in the mitochondria by GOT2, and Asp was transported into the cytoplasm where it could be converted into OAA by GOT1. Furthermore, OAA is converted into malate, which is then metabolized by malic enzyme 1 (ME1) to produce pyruvate. These reactions produced NAPDH potentially to maintain the cellular redox state (11, 58). Ziprasidone induced cell cycle arrest at the G1 phase and inhibited the phosphorylation of p38 and Erk, two key steps of MAPKs pathway (57). These findings indicate that targeting GOT1 in Gln metabolism in PADC may be a potential strategy for treating cancers.

GOT1 also plays a role in balancing the oxidative stress response induced by therapeutic drugs. Inhibition of GOT1 could regulate NADPH synthesis and then regulated ROS levels to counteract oxidative stress, which making colorectal cancer cells sensitive to 5-fluorouracil (12) and breast cancer cells sensitive to doxorubicin (15), enhancing anti-cancer effects. Additionally, knockdown of GOT1 decreased NADPH production and suppressed prostate cancer cell growth (18). Gln deprivation activated the expression of c-Myc, which enhanced the transcriptional expression of GOT1 and Nrf2. These molecules increased the glutathione synthesis from Glu and inhibited ferroptosis in hepatocellular carcinoma (HCC) cells. Interestingly, the researchers found that a combination of Gln deprivation and GOT1 inhibition caused fatal damage of HCC *in vitro* and *in vivo (* 47). These findings provide a novel strategy for improving the anti-cancer efficacy.

### 2.3 Regulation of glycolysis pathway

Emerging evidence has reported that GOT1 was also an important glycolytic enzyme that regulated the glycolysis pathway. In PDAC, shGOT1 cells displayed a consistent accumulation of glycolytic intermediates between the aldolase-catalyzed and pyruvate kinase-catalyzed steps of glycolysis, suggesting that GOT1 inhibition uniquely disrupted glycolysis pathways (13). Cancer cells obtained glucose through alternative metabolic pathways to meet their own requirements upon glucose deprivation. Inhibition of GOT1 sensitized the cancer cells to glucose deprivation, which was partially counteracted by metabolic intermediates downstream of GOT1 like OAA and phosphoenol pyruvate. GOT1 disruption in KRAS mutated cancer cells led to the up-regulated expression of the gluconeogenesis-pathway-gene glucose-6-phosphatase 3 *(G6PC3)* both during normal growth conditions and after glucose deprivation and the down-regulated expression of the glucose-controlling gene *BIP* after glucose deprivation, indicating GOT1’s roles in glucose metabolism (23). Non-coding RNA circGOT1 sponged miR-606 to promote GOT1, which induced glycolytic metabolism of esophageal squamous cell cancer cells, promoting cell proliferation (24). GOT1 inhibited glycolysis by interacting with pyruvate carboxylase and then inhibited malignant phenotypes of glioblastoma multiforme cells (59). GOT1 has also been reported to play roles in glycolysis in healthy cells. Sirt1 deletion in osteoblasts inhibited glycolysis by directly binding to and increasing the acetylation level of GOT1 to maintain bone homeostasis (60). Xu et al. found that GOT1 promoted the glycolytic programming and cytotoxic function of cytotoxic T lymphocytes via posttranslational regulation of HIF-1α protein, potentially by regulating the levels of α-KG (61). In early hypoxia, GOT1 participated in the regulation of glycolysis to maintain cells in a primed state that increased their chances of survival via sustaining cytoplasmic NAD^+^/NADH balance by sustaining flux through MDH1. In addition to its immediate contribution to glycolysis upon oxygen limitation, GOT1 activity contributed to α-KG turnover and thereby promoted HIF-1α stabilization (62, 63). Got1 also plays roles in diabetes. Got1 deletion in mouse β-cells impaired β-cell function by increased glycolysis and then led to phenocopying aging and diabetes (64).

### 2.4 Participation in immune cell function regulation

Asp synthesis is required for cancer cell proliferation. However, it is unclear whether Asp is limiting in healthy immune cells. Previous studies have indicated that conditional deletion of GOT1 from hematopoietic cells would be expected to increase Asp levels (10, 38, 39). Qi et al. found that mouse hematopoietic stem cells (HSCs) depended entirely on cell-autonomous Asp synthesis, which increased upon HSC activation (65). They established Got1 deficient mice and Got2 deficient mice, and found that Got1 deficiency increased Asp levels and HSC function while Got2 deficiency reduced Asp levels and HSC function during hematopoietic regeneration. While Got1 deficiency and Got2 deficiency had opposite effects on Asp levels, both would be expected to disrupt the MAS. Deletion of both Got1 and Got2 was lethal for HSCs because that they were unable to synthesize nucleotides or other products producing energy (65). Ma et al. identified Got1 was a regulator of Gln-dependent Asp production in T cells and was required for CD8^+^ T cell responses *in vivo (* 66). CD8^+^ T cells expressing Got1-targeting shRNA displayed reduced Gln-derived Asp and slight reduced cell expansion. *In vivo* experiments, they observed a marked reduction of percentage and number of antigen-specific CD8^+^ T cells responding to LmOVA infection. Moreover, Got1 silencing altered the CD8^+^ T cell effector response, as evidenced by an overall lower percentage and number of IFN-γ-producing CD8^+^ T cells in the spleens of LmOVA-infected animals, indicating a critical function for Got1 in mediating the expansion of CD8^+^ effector T cells *in vivo (* 66). Recently published work by Xu et al. established similar requirements for GOT1 in mediating T cell-mediated anti-tumor responses. Xu et al. found that GOT1 was upregulated in effector CD8^+^ T cells, which promoted their differentiation and function by maintaining intracellular redox balance and serine-mediated purine nucleotide biosynthesis. Additionally, GOT1 promoted the glycolytic programming and cytotoxic function of cytotoxic T lymphocytes via posttranslationally regulating HIF-1α expression, potentially by regulating the levels of α-KG (61). The MAS including GOT1 detoxified ammonia in exhausted T cells by producing 2-ketoglutarate (2-KG) (67). CD8^+^ T cells expressed GOT1 during chronic infections to execute antiviral responses and to decrease the concentration of ammonia to promote the assimilation of free ammonia and cell survival by producing 2-KG. GOT1 promoted effector T cell formation in acute lymphocytic choriomeningitis virus infection dependent on the traditional function of GOT1 in maintaining the NAD^+^/NADH ratio. They also indicated that GOT1 deficiency influenced the transcriptional profiles and epigenetic landscapes of CD8^+^ T cells via decreasing the expression levels of genes involved in demethylation, such as *Kdm6b* and *Tet1 (* 67). GOT1 could also regulate adipocyte differentiation by altering the NADPH content (68). Macrophages play a central role in host innate immune response defending against pathogens. A study has shown that Got1 was dispensable for M2 macrophage differentiation and did not influence the onset of lipopolysaccharides-induced immune tolerance in macrophages (69).

### 2.5 Participation in epigenetic regulation

Metabolism has been shown to integrate with epigenetics and transcription to modulate cell fate and function (70–72). Xu et al. identified a small molecule aminooxyacetate (AOA) reprogramed T_H_17 differentiation toward iTreg cells and Got1 was the main target of AOA during T_H_17 cell differentiation. Knock-down of Got1 in differentiation T_H_17 cells inhibited T_H_17 cell differentiation and reciprocally increased iTreg cell differentiation. T_H_17 cell differentiation was regulated mainly via Got1-dependent increased transamination leading to elevated 2-hydroxyglutarate (2-HG) level in differentiating T_H_17 cells. And accumulating 2-HG resulted in hypermethylation of *FOXP3* gene locus and inhibited *FOXP3* transcription, which was essential for fate determination towards T_H_17 cells (73). The study of Xu et al. suggested an important mechanistic link of Got1 activity in the fate determination of T_H_17 cell differentiation by an epigenetic mechanism. GOT1 activity could be enhanced by tumor suppressor SIRT5 deletion via facilitating GOT1’s lysine acetylation to regulate the non-canonical Gln and glutathione metabolism, and then promoted tumorigenesis of Kras-induced pancreatic cancer (45). SIRT1 inhibited GOT1 enzyme activity by catalyzing its lysine deacetylation in osteoblasts to regulate osteoblast metabolism (60). Future work into the regulatory underpinnings of GOT1 can provide a better understanding for differential metabolic activities and dependencies.

## 3 Emerging GOT1-related pathways in cancer

The expression of GOT1 can be regulated by non-coding RNAs including circular RNAs (circRNAs), microRNAs (miRNAs), and long non-coding RNAs (lncRNAs), and then affecting cell proliferation and metabolism (46, 48) (**Table 1**). In addition, lncRNAs and circRNAs usually affect cancer cell proliferation via sponging miRNAs targeted GOT1 directly. For example, circ-MBOAT2 silencing downregulated the expression of GOT1 via sponging miR-433-3p, modulating the tumor development of PDAC (51). In non-small cell lung cancer, hsa_circRNA_103809 sponged miR-337-3p to upregulate the expression of GOT1, affecting the cisplatin-resistance and cell growth (50). CircGOT1 was generated from exon 8 of its host gene GOT1 via back-splicing and then promoted GOT1 expression. CircGOT1 promoted the expression of GOT1 via sponging miR-606 to exert carcinogenesis including promoting tumor growth, migration, and glycolytic metabolism of esophageal squamous cell cancer cells (24). Furthermore, miR-9-5p could directly bind to the 3’-untranslated region (3’UTR) of GOT1 and then inhibited the expression of GOT1, which stunted the proliferation, invasion, Gln metabolism, and redox homeostasis of pancreatic cancer cells (48). Additionally, exosome-derived lncRNA NEAT1 functioned as a ceRNA of miR-9-5p to facilitate the expression of TFRC and GOT1, and then exacerbated ferroptosis of sepsis-associated encephalopathy (52). lncRNA TMPO-AS1 was highly expressed in HCC cells and expedited HCC progression via promoting cell proliferation, stemness as well as suppressing cell apoptosis. Molecular mechanism showed TMPO-AS1 functioned as a molecular sponge for miR-429 and GOT1 served as a downstream target gene of miR-429 in HCC. miR-429 could directly bind to GOT1 and negatively regulated the expression of GOT1 while TMPO-AS1 positively regulated the expression of GOT1. Additionally, GOT1 overexpression reversed the inhibition effects of TMPO-AS1 deficiency on HCC progression, indicating that the TMPO-AS1/miR-429/GOT1 axis may be an underlying treatment strategy for HCC (49).

**Table 1:** Non-coding RNAs regulate the expression of GOT1 in different tumors.

Tumor	Targeting pathway	Functional roles	Expression	References
Esophageal squamous cell cancer	circGOT1/miR-606/GOT1	promoted cell proliferation, migration, aerobic glycolysis, and cisplatin resistance	Up	(24)
Pancreatic cancer	miR-9-5p/GOT1	stunted proliferation, invasion, glutamine metabolism, and redox homeostasis.	Down	(48)
Hepatocellular Carcinoma	LncRNA TMPO-AS1/ miR-429/GOT1	aggravated cancer progression via promoting cell proliferation, stemness as well as suppressing cell apoptosis	Up	(49)
Non-small cell lung cancer	hsa_circRNA_103809/ miR-377-3p/GOT1	increased susceptibility of cisplatin-resistant non-small cell lung cancer cells to cisplatin	Up	(50)
Pancreatic cancer	Circ-MBOAT2/miR-433-3p/GOT1	inhibited cell proliferation, migration, invasion, and glutamine metabolism	Up	(51)
Melanoma	miR-9/GOT1	suppressed ferroptosis of melanoma cells	Down	(74)

GOT1 is also a potential target of transcriptional regulators. Paralogous transcriptional regulators TAZ and YAP are considered as central factors in cancer biology and they play key roles in cell proliferation, survival, and cell fate determination (75). TAZ/YAP reprogram cellular energetics to promote the dependence of breast cancer cell growth on exogenous Gln, and TAZ/YAP induced GOT1 and phosphoserine aminotransferase (PSAT1) expression to promote the conversion of Gln to α-KG, and then to support cell growth (14). Additionally, targeting metabolic vulnerability to block of transamination of GOT1 using AOA suppressed the growth of breast cancer cells in a TAZ/YAP-dependent manner (14). These findings indicate more remains to be investigated regarding the role of GOT1 in transcriptional regulation in cancers.

Recent studies have indicated that GOT1 played critical roles in ferroptosis. For instance, in melanoma, miR-9 targeted GOT1 directly, inhibiting the expression of GOT1, which suppressed ferroptosis of melanoma cells (76). In multiple myeloma, GOT1 expression was inhibited by shikonin, enhancing ferroptosis in multiple myeloma cells by promoting the release of autophagic labile iron (21). Additionally, GOT1 inhibition increased labile iron availability through autophagy, enhancing the activity of ferroptosis and impaired proliferation and promoted cell death of pancreatic cancer cells (74). The expression of GOT1 was also up-regulated in pancreatic cancer cell-derived exosomes, promoting cancer cell proliferation, migration, and invasion (77–79). Experimental evidence found that pancreatic cancer cell-derived exosomes enriched with GOT1 inhibited cellular ferroptosis to promoted pancreatic cancer cell progression through mechanisms like upregulating CCR2 expression and activating the Nrf1/HO2559-1 pathway (80). These findings highlight the importance of GOT1 regulation through ferroptosis in cancer treatment strategies, and the necessity to study how genetic and environmental factors influence ferroptosis susceptibility *in vivo*.

## 4 Conclusion

Cellular metabolic reprogramming is a hallmark of cancer, serving as a therapeutic target to overcome limitations in current therapies of cancers. The body of this work involving GOT1 highlights several key points in cancer metabolism. First, cancer cells exhibit dynamic metabolism to support abnormal growth and functions. Second, the metabolic pathways utilized by cancer cells are influenced by various factors such as endogenous signals, tumor microenvironment, nutritional composition, external signals, and oncogenes. Third, using appropriate disease models in the suitable context and environment is crucial for identifying therapeutic targets in cancer metabolism.

A major challenge in targeting cancer metabolism lies in the rapid adaptability of cancer cells to adjust their metabolic pathways (81). Cancer cells can quickly modify their metabolic strategies to utilize available metabolic intermediates from nutrient-deficient environments, synthesizing the energy necessary for their growth. As a component of MAS, the inhibition of GOT1 leads to a decrease in NADPH production. NADPH is essential for multiple metabolic pathways, including glycolysis, and its reduction results in decreased flux into these metabolic routes. Under such circumstances, tumor cells activate a specific metabolic pathway known as non-canonical Gln metabolism replacing the canonical Gln metabolism (82) to meet their energetic demands. In PDAC, GOT1 inhibition resulted in Gln deprivation, dimethyl α-KG did not restore growth upon Gln deprivation, whereas the combination of α-KG and a non-essential amino acid mixture rescued proliferation, suggesting PDAC cells metabolize Gln in a model that is different from canonical models. Gln deprivation led to decreased Gln-derived malate, and Gln-derived OAA converted by GOT1 was metabolized into malate. Malate was utilized by ME1 to generate NADPH, maintaining cell growth (10). In PDAC cells, tumor Asp availability was maintained by both *de novo* synthesis and alternative extracellular sources in the absence of GOT1/GOT2 *in vivo (* 83). Researchers found that PI3K/mTORC2 pathway promoted GOT1 expression via targeting hypoxia-inducible factor HIF-2α, and then activated the non-canonical Gln metabolism *in vitro* and *in vivo*, promoting progression of PDAC (46). GOT1 also plays an important role in an acidic tumor microenvironment. In pancreatic cancer, in response to chronic acidosis stress, the expression of GOT1 was increased in cancer cells to fuel oxidative metabolism by enhancing the non-canonical Gln metabolism (84). Cancer cells adapt to nutrient-deprived tumor microenvironment during progression via adjusting the level and function of metabolic enzymes. Inhibition of GOT1 significantly weakened HCC cell proliferation under high glucose conditions, while silencing of glutamate dehydrogenase 1(GDH1) did not take effect. Deletion of GDH1 impaired HCC cell proliferation under low glucose conditions, yet knock-down of GOT1 did not take effect. Moreover, GDH1 expression was elevated in glucose-poor HCC tissues while GOT1 expression was decreased. However, inhibiting Gln-dependent transaminases including GOT2, GPT-1, GPT-2, and PSAT-1 had essentially no impact on proliferation of cells no matter under high or low glucose conditions, suggesting that liver cancer cells maintained survival under different nutritional conditions through the metabolic flexibility of their Gln-related enzymes (63). Collectively, these studies indicate that in the absence of GOT1, cells possess both endogenous and exogenous compensatory mechanisms that allow cancer cells to survive. Thus, GOT1 may be served as a key source to fuel the rewired metabolic pathways.

Although metabolism-targeted therapy is not yet standard therapy for many cancers, several experimental and clinical trials targeting altered metabolism are currently underway. For example, WZB117, a specific GLUT1 inhibitor, could inhibit the tumor-initiating capacity of the cancer stem cells *in vitro* and inhibit tumor initiation *in vivo (* 85). Adapalene, an approved drug clinically used in the therapy of acne vulgaris, could selectively inhibit the activity of GOT1 in a non-competitive manner and further suppress the proliferation of ovarian cancer ES-2 cells (86). AOA, an inhibitor of pyridoxal 5-phosphate-dependent enzymes, could also inhibit the activity of GOT1, causing amino acid deprivation (87, 88). Although numerous small-molecule drugs have been proven to inhibit the activity of GOT1 in a competitive or non-competitive manner (23, 57, 79, 86, 89–91), further efforts are still required for the development of specific inhibitors targeting GOT1. Moreover, none of these GOT1 inhibitors have progressed to clinical trials yet, necessitating further research to develop effective GOT1 inhibitors for cancer treatment. Considerable work remains to translate the basic research on GOT1-targeted drug therapy into clinical applications.

While attempts to treat cancers by targeting cellular metabolism have achieved some success, the development of drug resistance is still a significant challenge in the design of metabolic targeted therapies. It is worth noting that metabolic networks possess remarkable plasticity, allowing them to reconnect and circumvent targeted treatments. Furthermore, certain tumor cells exhibit heterogeneous metabolic subtypes, which exhibit varying prognoses and metabolic susceptibilities (92–94). Consequently, these factors should be thoroughly considered when designing effective metabolic targeted therapies. As research progresses, it is urgent to develop specific and effective drugs targeting metabolism. In the near future, more efficient metabolism-targeting drugs and treatment regimens are expected to develop to improve patient outcomes and extend survival, yielding clinical benefits for patients.
